# The reliability and validity for Japanese type 2 diabetes patients of the Japanese version of the acceptance and action diabetes questionnaire

**DOI:** 10.1186/s13030-018-0129-9

**Published:** 2018-08-02

**Authors:** Junichi Saito, Wataru Shoji, Hiroaki Kumano

**Affiliations:** 1 Graduate School of Human Science, Waseda University, 2-579-15, Mikajima, Tokorozawa, Saitama, 359-1192 Japan; 2 Graduate School of Medicine, Tohoku University, Sendai, Japan; 3 Faculty of Human Science, Waseda University, Tokorozawa, Japan

**Keywords:** Acceptance and commitment therapy, Questionnaire development, Type 2 diabetes

## Abstract

**Background:**

The purpose of this study was to determing which psychological traits of Japanese type 2 diabetes patients would provide reliability and validity to the Japanese version of the Acceptance and Action Diabetes Questionnaire (AADQ-J).

**Methods:**

Various questionnaires were administered to type 2 diabetes patients who were registered on the database of the research service provider; data from a total of 600 patients (mean ± SD age was 57.50 ± 9.87 years, female 21.83%) were analyzed.

**Results:**

Three items were excluded because of psychometric concerns related to the original 11-item AADQ. Confirmation factor analyses revealed that the eight-item version demonstrated the best indicators of a goodness of fit. The questionnaire showed adequate internal consistency. The questionnaire demonstrated high measurement accuracy in broad trait values by the test information function of Item Response Theory. The questionnaire showed stronger positive correlations with self-care activities and HbA1c than with diabetes distress and depressive mood.

**Conclusions:**

The eight-item Japanese version of AADQ has reliability and validity for type 2 diabetes patients.

## Background

Globally, the prevalence of type 2 diabetes is increasing; currently, 422 million adults are living with diabetes. Further, the number of sufferers expected to die from complications of the disease is predicted to double between 2005 and 2030 [1]. Diabetes is mainly classified into type 1 diabetes and type 2 diabetes depending on pathogenesis. The main cause of type 1 diabetes is the lack of insulin action resulting from the destruction and loss of *β* pancreatic islet cells, which promote the synthesis and secretion of insulin. Therefore, “insulin therapy” becomes the main treatment method. On the other hand, type 2 diabetes developes from a combination of multiple genetic factors, which include predisposition to insulin secretion decrease and insulin resistance, ageing, and behavioral and psychosocial factors such as overeating (especially high dietary fat intake), lack of exercise, obesity, and stress. Type 2 diabetes is a representative life-style related disease, and self-care activities (mainly diet and exercise) will be the essential options for its treatment. In the treatment of type 2 diabetes, self-care activities are required for patients to a much greater degree than other physical disorders. In addition, psychological distress associated with types 2 diabetes may cause concurrent psychiatric disorders such as depression. The rate of depression in the non-type 2 diabetes population is 9.8%, while the rate in the type 2 diabetes patient populations is 17.9%; thus, the prevalence rate of depression is significantly higher for type 2 diabetes patients than for the non-type 2 diabetes population [2]. Physiologically, depressive symptoms not only suppress insulin action, but may also lead to inadequate self-care activities. This, in turn, may result in an increase in the blood glucose level [3]. Therefore, psychological therapies for diabetes patients have been conducted so as to alleviate psychological distress.

Studies have indicated that although psychological therapies, including cognitive behavior therapy (CBT), alleviate psychological distress and improve the blood glucose level; these therapies do not have a long-term effect [4, 5]. In psychological therapies including CBT, patients are taught to control unpleasant thoughts and feelings. However, diabetes treatment requires lifetime adherence and thus, ongoing management of unpleasant thoughts and feelings that are related to diabetes may not be a realistic strategy [6]. Furthermore, as per research, psychological therapies have not been practically employed because of the time and effort needed to do so [7].

Recently, Acceptance and Commitment Therapy (ACT) has been recognized as an alternative approach to other psychological therapies [8]. In ACT, instead of controlling unpleasant thoughts and feelings, acceptance is emphasized. This approach involves becoming aware of unpleasant thoughts and feelings without attempting to change the frequency and form thereof. ACT is unique in its efforts to help one have a better quality of life by accepting unpleasant thoughts and feelings related to diabetes as well as making a commitment to one’s values rather than deciding on diabetes treatment as a goal.

One study found that, in comparison to patients who only received diabetes education, patients in the ACT intervention condition were more likely to report superior self-care activities and have glycated hemoglobin (HbA1c) values in the target range of under 7% after three months [6]. Mediational analysis showed that an increase in acceptance as assessed by the Acceptance and Action Diabetes Questionnaire (AADQ) mediated improvement in self-management and HbA1c values. Because of the intervention effect of a one-day education workshop, which was indicated by this study, introducing psychological therapeutic interventions in the diabetes healthcare system on reasonable terms was a possible expectation.

However, a study criticized the lack of reports on the development of AADQ; thus, its psychometric methodical traits are unclear [9]. In fact, the original version consisted of one 11-item factor. However, on the basis of I-T correlation analysis, three items were excluded in a one paper [10]. Further, as the result of factor analysis, two items were excluded because of bidimensionality. Eventually, the resulting single 6-item factor showed good item properties and reliability. It is noteworthy that the sample was collected at a tertiary referral center where there was a high prevalence of type 1 diabetes, which suggests that this sample may have differed from community samples [10]. We assume Type 1 and Type 2 diabetes have different AADQ factor structures as a result of the differences in pathogenesis and treatment methods. The aim of the present study was to examine the psychological traits of the Japanese version of AADQ, AADQ-J, in an effort to develop a reliable and valid measure for use with Japanese type 2 diabetes patients.

## Methods

### Procedure

To collect data from a wide range of community samples, we conducted an online survey with the assistance of a marketing research service provider instead of giving it at medical institutions. We obtained valid responses from 300 sufferers in the first sample, sample 1, and a further 300 individuals in the second sample, sample 2, from approximately 8216 Japanese type 2 diabetes patients who were registered on the database of the research service provider.

### Demographics

Sociodemographic information pertaining to age, sex, complication, and treatment status were obtained through self-report on the respective questionnaires.

### Diabetes acceptance

The Acceptance and Action Diabetes Questionaire (AADQ) has 11 items; it measures acceptance of diabetes-related thoughts and feelings, and the degree to which they perform valued action [6]. An example of an item includes, “I avoid thinking about what diabetes can do to me.” All the items are reverse scored with the exception of one item: “I have thoughts and feeling about being diabetic that are distressing.” The items are rated on a 7-point Likert scale, ranging from 1 = never true to 7 = always true.

The following four steps were employed in the translation process. First, the second author, Shoji, W., a clinical psychologist, translated all of the original AADQ items from English into Japanese. This initial translation was then checked by the last author, Kumano, H., an expert ACT therapist. Any differences in the meaning or clarity of the translations of these authors were discussed and resolved by means of consensus. In the third step, the revised Japanese version (AADQ-J) was back-translated from Japanese into English by a native English speaker who is also fluent in Japanese. Finally, the back-translation was checked and approved by the developer of the original AADQ.

### Diabetes self-management

The Summary of Diabetes Self-Care Activities Measure (SDSCA) comprises 17 items that measure the frequency of performing diabetes self-care activities during the previous seven days [11]; these activities include diet, exercise, blood glucose testing, foot care, and tobacco use. In this study, the subscales of diet and exercise of the Japanese version of SDSCA were employed [12]. For each question, the respondent marks the number of days the indicated behavior was performed on an 8-point Likert scale.

### Diabetes-specific distress

The Problem Areas in Diabetes Treatment Satisfaction Questionnaire (PAID) measures diabetes-specific distress [13]; it comprises 20 items. In this study, the Japanese version of PAID [14] was employed. The respondent rated each item on a 5-point Likert scale, ranging from 0 = not a problem to 4 = serious problem.

### Quality of life

The Short Form-8 Health Questionnaire (SF-8) [15] is an eight-item questionnaire that measures physical and mental health-related quality of life. In this study, the Japanese version of SF-8 [16] was utilized. Data from SF-8 is represented as both a physical component score and a mental component score.

### Depressive symptoms

The Center for Epidemiologic Studies Depression Scale (CES-D) measures depressive symptoms in the general population; it comprises 20 items [17]. In this study, the Japanese version of CES-D [18] was employed. Respondents are required to rate each item on a 4-point Likert scale, ranging from 0 = rarely or none of the time to 3 = most or all of the time.

### Hemoglobin A1c

The Hemoglobin A1c (HbA1c) levels of the patients [non-diabetic range 23.5–43.2 mmol/mol (4.3–6.1%)] were self-reported data. HbA1c is the most common assessment of glycemic control. The HbA1c level is an indication of average blood glucose over the previous one to 2 months.

### Statistical analysis

Item Response Theory was applied to examine the characteristics of the items. The AADQ-J uses a 7-point Likert scale and, accordingly, a graded response model was selected. We performed exploratory and confirmatory factor analyses so as to consider the factor structure of AADQ-J. Data from sample 1 were employed for these analyses.

Reliability was determined by the internal consistency of Cronbach’s α and the test information curve from Item Response Theory. Criterion-related validity and discriminant validity were also examined. Data from sample 2 was employed for these analyses. Further, all analyses were performed by utilizing R version 3.4.3.

## Results

### Sample characteristics

Sample 1 consisted of 300 patients; of these, 101 (33.67%) were female. The patients’ mean ± SD age was 56.68 ± 10.06 years. Of the 300, 89 (29.67%) were untreated or discontinued treatment. The prevalence of diabetes complications was as follows: retinopathy, 8.33%; neuropathy, 2.33%; and nephropathy, 4.33%. Sample 2 included 300 patients; 32 (9.36%) were female. The patients’ mean ± SD age was 58.33 ± 9.68 years. Of the 300, 90 (30.00%) were untreated or had discontinued treatment. The prevalence of diabetes complications was as follows: retinopathy, 10.33%; neuropathy, 7.00%; and nephropathy, 3.33%.

### Item response theory

Item discrimination (*a*) and difficulty (*b*) were estimated for each item of the AADQ-J by employing Item Response Theory (Table 1). Every item was shown to have moderate discrimination [19] (values from 0.01 to 0.24 are considered very low, 0.25–0.63 low, 0.65–1.34 moderate, 1.35–1.69 high, and > 1.7, very high). On the contrary, with reference to difficulty, items 3 and 6 showed negative values at every level, indicating that the difficulty of two of the items was extremely low. For this reason, these two items were excluded from the original Acceptance and Action Diabetes Questionnaire.

**Table 1:** Item Response Theory parameter estimates for the Japanese version of Acceptance and Action Diabetes Questionnaire

Items		Item parameter estimates^a^						
		*a*	*b*_1_	*b*_2_	*b*_3_	*b*_4_	*b*_5_	*b*_6_
1	I try to avoid reminders of my diabetes.	0.97	−2.86	−2.36	−1.87	−1.06	0.20	0.73
2	I have thoughts and feelings about being diabetic that are distressing.^b^	−0.76	1.76	1.31	0.11	−0.93	−1.53	−2.32
3	I do not take care of my diabetes because it reminds me that I have diabetes.	1.00	−3.13	−2.65	− 2.37	−1.92	− 1.09	− 0.62
4	I eat things I shouldn’t eat when the urge to eat them is overwhelming.	0.87	−2.25	−1.65	−0.64	0.86	1.45	2.18
5	When I have an upsetting feeling or thought about my diabetes, I try to get rid of that feeling or thought.	0.98	−2.76	−2.50	−1.73	−0.68	0.54	1.09
6	I avoid taking or forget to take my medication because it reminds me that I have diabetes.	0.91	−3.68	−3.05	−2.73	−2.02	−1.22	−0.64
7	I avoid stress or try to get rid of it by eating what I know I shouldn’t eat.	1.03	−2.44	−1.81	− 1.27	−0.12	0.73	1.29
8	I often deny to myself what diabetes can do to my body.	1.14	−2.57	−2.11	−1.59	−0.87	0.08	0.66
9	I don’t exercise regularly because it reminds me that I have diabetes.	0.72	−3.41	−2.49	−1.88	−1.19	0.04	0.52
10	I avoid thinking about what diabetes can do to me.	0.89	−2.43	−1.60	−1.08	−0.41	0.61	1.05
11	I avoid thinking about diabetes because someone I knew died from diabetes.	0.88	−3.53	−2.65	−2.44	−1.83	−0.48	0.14

^a^Each of the *b* parameters corresponds to a probability = 0.5 of choosing the response that is + 1 from the subscript. The *a* parameter is the slope at the location of all b parameters and corresponds to the item’s ability to discriminate between individuals of different trait levels.^b^All items are reverse scored except Item 2

### Factor structures

At first, we proceeded with the minimum average partial method to explore and construct a statistically justifiable factor structure for the nine-item scale. The results of the minimum average partial method showed one factor structure for the nine-item scale. To confirm the structure of the nine-item scale, factor analysis (maximum-likelihood method) was conducted. Results revealed that item 2, the only order item in the original version, demonstrated negative values in factor loading. As a result, it was excluded from the measurement when logical consistency was considered. Factor analysis was performed again with the remaining eight items. The proportion of variance explained was 42.50%.

To test the suitability of the structure suggested by exploratory factor analysis, we conducted confirmatory factor analysis. In addition, so as to compare the eight-item scale, we conducted confirmatory factor analysis with the original eleven-item scale and the six-item scale [10]. The resulting eight-item scale demonstrated good indicators of goodness of fit (Table 2).

**Table 2:** Results of Confirmatory Factor Analysis of the Japanese version of Acceptance and Action Diabetes Questionnaire

Items		Factor loading		
		original 11-item version	Schimmit’s 6-item version	8-item version
1	I try to avoid reminders of my diabetes.	0.62	0.67	0.56
2	I have thoughts and feelings about being diabetic that are distressing.^b^	− 0.55		
3	I do not take care of my diabetes because it reminds me that I have diabetes.	0.50	0.53	
4	I eat things I shouldn’t eat when the urge to eat them is overwhelming.	0.55		0.54
5	When I have an upsetting feeling or thought about my diabetes, I try to get rid of that feeling or thought.	0.67	0.69	0.64
6	I avoid taking or forget to take my medication because it reminds me that I have diabetes.	0.46	0.49	
7	I avoid stress or try to get rid of it by eating what I know I shouldn’t eat.	0.63		0.62
8	I often deny to myself what diabetes can do to my body.	0.71	0.67	0.76
9	I don’t exercise regularly because it reminds me that I have diabetes.	0.53		0.53
10	I avoid thinking about what diabetes can do to me.	0.61	0.58	0.66
11	I avoid thinking about diabetes because someone I knew died from diabetes.	0.57		0.57
Models		Indicators of goodness of fit		
		CFI	RMSEA	SRMR
original 11-item version		0.78	0.13	0.08
Schimmit’s 6-item version		0.79	0.20	0.09
8-item version		0.84	0.12	0.07

^b^All items are reverse scored except Item 2

### Reliability

**Fig. 1:**
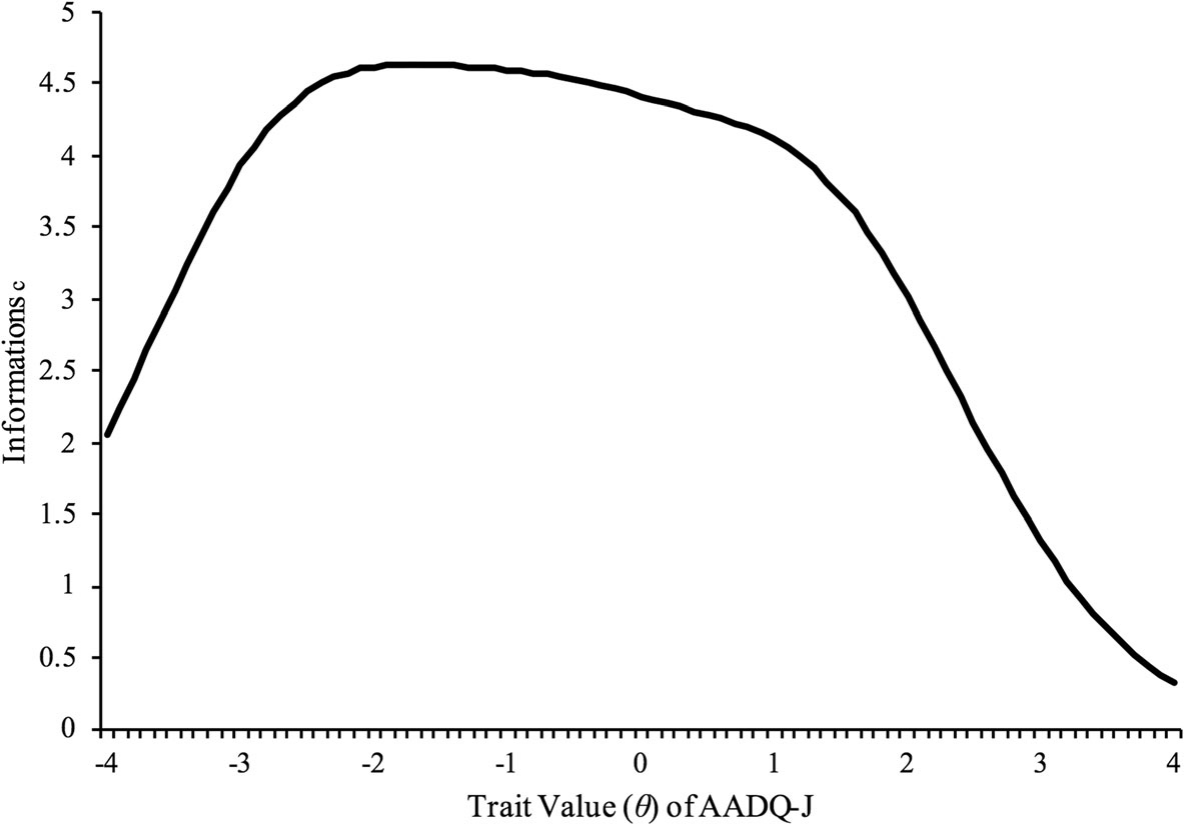
Information for the Japanese version of Acceptance and Action Diabetes Questionnaire across trait estimates. ^c^ Information is determined for each item at each trait value at each response threshold, where there are k-1 response thresholds (k = total number of response options). Total information is the sum of information across all trait values and all response options for each item. In this figure, total scale information is represented

When examining the internal consistency of the AADQ-J, Cronbach’s α showed 0.80. When considering the test information function from Item Response Theory, Trait values (*θ*) showed high values, between − 3 and 2; this demonstrated high measurement accuracy in a wide range of Trait values (*θ*) (Fig. 1).

### Validity

To evaluate criterion-related validity, the correlations between the AADQ-J and SDSCA, SF-8 and HbA1c were calculated. Regarding the self-care activities, a moderate positive correlation was shown with diet in the SDSCA, as was a weak positive correlation with exercise. Regarding the physical and mental quality of life, a weak positive correlation was shown with the physical component of SF-8, as was a moderate positive correlation with the mental component. Regarding the glucose level, a weak negative correlation was shown with HbA1c.

To evaluate discriminant validity, the comparative associations between AADQ-J, PAID, and CES-D with other measures were calculated. Both diabetes distress in PAID and depressive mood in CES-D were more strongly related to mental health-related quality of life in SF-8 than in AADQ-J. In contrast, the AADQ-J showed stronger positive correlations with self-care activities in SDSCA and a negative correlation with HbA1c than diabetes distress and depressive mood (Table 3). Moreover, we performed Welch’s *t* test with the treatment condition (treatment group and untreated or discontinued treatment group) as independent variables and the AADQ score as the dependent variable to consider the difference between treatment conditions. The results showed statistically significant differences at the 1% level (treatment group (*N* = 210; diabetes duration 10.79 ± 8.23 years, HbA1c 6.82 ± 0.68) average 42.83 SD 6.10: untreated or discontinued treatment group (*N* = 90; diabetes duration 8.50 ± 8.91 years, HbA1c 7.06 ± 1.03) average 39.44 SD 9.45, *t* = 3.13, *p* = .00, Hedges’ *g* = .47).

**Table 3:** Correlation Coefficients of the AADQ-J with Other Measures, Means and Standard Deviations (*SD*)

	AADQ-J	PAID	CES-D	SDSCA_Diet	SDSCA_Ex	SF-8_PCS	SF-8_MCS	HbA1c
AADQ-J	–	−0.44^**^	−0.41^**^	0.37^**^	0.24^**^	0.29^**^	0.32^**^	− 0.21^**^
PAID	–	–	0.49^**^	− 0.14^*^	0.04	− 0.25^**^	− 0.37^**^	0.19^**^
CES-D	–	–	–	− 0.30^**^	− 0.15^**^	− 0.33^**^	− 0.68^**^	0.06
*Means*	41.82	43.98	16.00	22.35	7.40	46.58	44.78	6.81
*SD*	7.42	14.40	9.21	6.71	4.09	7.77	7.86	0.82
*Sample size*	300	300	300	300	300	300	300	211

^*^ *p* < 0.05, ^**^ *p* < 0.01

## Discussion

The aim of the present study was to examine the psychological traits relevant to the reliability and validity of the Japanese version of AADQ for Japanese patients with type 2 diabetes.

By adapting Item Response Theory to each item of the AADQ, moderate discrimination and difficulty were indicated for most items. However, the difficulty of items 3 and 6 were extremely low. Both items were reversal items; clarifying the low difficulty most have denying these items (item 3: “I do not take care of my diabetes because it reminds me that I have diabetes;” and item 6: “I avoid taking or forget to take my medication because it reminds me that I have diabetes”). In a previous study [10], these two items were not excluded; factor loading by means of factor analysis was revealed. A reason for this difference is possibly that type 1 diabetes patients comprised 70% of the sample in the study [10] while in the current study we only focused on type 2 diabetes patients. In contrast to type 1 diabetes patients who need insulin injections, many type 2 diabetes patients require only oral medications in addition to self-care activities. It has been demonstrated that the psychological distress of type 1 and type 2 diabetes patients differ due to differences in clinical condition and treatment method [20]. Item 6 would understandably be denied by many type 2 diabetes patients whose treatment is mainly based on oral medication; this item focuses on not only oral medication but also on insulin injection and their continuation. Also, from the viewpoint of social desirability among Japanese patienst, it might have been hard for them to affirm item 3, which means that the patients confess that they avoid commitment to their diabetes treatment.

The results of exploratory factor analysis showed that the factor loading of item 2 in AADQ-J, the only order item from the original version and that had a negative factor loading after reversing the item scores, lacked logical consistency. Cultural differences in the understanding of the content of the item, differences in expression resulting from the process of translation, and psychometric influences because of being the only order item are possible reasons for this phenomenon. The confirmatory factor analysis results also indicated that these three items should be excluded from the total score.

The Cronbach’s α of 0.80 proved that the AADQ-J consisting of eight items had adequate internal consistency. The results of the test information function of Item Response Theory revealed that this measurement can yield an accurate measurement for a wide range of patients; from patients who have stopped treatment and whose acceptance levels are predicted to be low to patients who have continued with treatment and whose acceptance levels are predicted to be high.

We investigated the relation between AADQ-J and self-care activities, quality of life, and HbA1c to assess criterion related validity. In addition, we investigated the discriminant validity between AADQ-J and diabetes related distress and depressive symptoms. The results concurred with those of another study [10]. AADQ-J was more related to increases in self-care activities and a reduction in HbA1c level, while diabetes-related distress and depressive symptoms were more related to mental health-related quality of life. It indicates that clinical practice should focus on depressive symptoms and diabetes-related distress [21]. However, it should be noted that we have also demonstrated the clinical significance of focusing on acceptance as a new perspective.

The limitations of this study and recommendations for further studies are as follows. Firstly, because the current study was carried out by Internet survey, items such as sociodemographic information and HbA1c were based on the participant’s self-report. This may be the reason for the low rate of diabetes complication. Secondly, over 90% of the participants in sample 2 were male; however, we were able to confirm that there was no gender difference in AADQ average score (male: 41.83 ± SD 0.45, female: 41.68 ± SD 1.31). Thirdly, a possibility of a difference in the factor structure between type 1 and type 2 diabetes patients was indicated. The number of female participants should be increased in future studies. In addition, the clinical usefulness of this measurement by involving both type 1 and type 2 diabetes patients should be examined in future studies.

## Conclusions

We investigated the psychometric properties of the Japanese version of AADQ in an effort to develop a reliable and valid inventory for Japanese type 2 diabetes patients. The eight-item Japanese version of AADQ was shown to be reliable and valid for these patients. The result of the test information function from Item Response Theory revealed that an accurate measurement can be made for a wide range of patients.

## Abbreviations

AADQ-J: Japanese version of Acceptance and Action Diabetes Questionnaire
ACT: Acceptance & commitment therapy
CBT: Cognitive behavior therapy
CES-D: The Center for Epidemiologic Studies Depression Scale
CFI: Comparative fit index
PAID: The problem areas in diabetes treatment satisfaction questionnaire
RMSEA: Root mean square error of approximation
SDSCA_Diet: The summary of diabetes self-care activities measure subscale of diet
SDSCA_Ex: The summary of diabetes self-care activities measure aubscale of exercise
SF-8_MCS: The short form-8 health questionnaire as a mentalcomponent score
SF-8_PCS: The short form-8 health questionnaire as a physical component score
SRMR: Standardized root mean-square residual
